# A Concise Analysis of Professors Hufeland and Kant's Treatise on the Art of Prolonging Life

**Published:** 1799-05

**Authors:** 


### *A conciſe Analyſis of Profeſſors* Hufeland *and* Kant*'s Treatiſe on the Art of Prolonging Life.*

Dr. Hufeland, of Jena, had ſent to the Pruſſian philoſopher Kant a copy of his '*Macrobiotick*,' or the art of prolonging life (which has lately been tranſlated into Engliſh), and requeſted his opinion of that performance. The treatiſe now under conſideration contains Kant's anſwer. It is intitled, '*On the faculty of the mind to conquer, by the ſimple act of the will, the ſenſations produced by diſeaſes.*' It has been lately publiſhed by Dr. Hufeland, with his own obſervations on that ſubject.

Kant declares, that he is not an advocate for keeping the head and feet warm. The Ruſſians, in their ſevere climate, adopt the contrary rule, which they even extend to the breaſt; yet they are not liable to catch cold. Although it may be more agreeable, it is a prejudicial cuſtom to bathe the feet in warm water; for this practice produces a relaxation of the blood-veſſels in the extremities, which is not unfrequently the cauſe of incurable diſorders of the legs and feet. It is however adviſable, during the rigorous ſeaſon, carefully to guard the abdomen againſt cold.—Too much ſleep he conſiders to be an enemy to longevity; it is apt to produce weakneſs and epilepſy in nervous habits; and, inſtead of affording nouriſhment, has a directly contrary effect; as has every degree of exceſſive care of the body. He juſtly conſiders equanimity of mind as a great means of preſerving the body, and leading to old age. He obſerves, that having himſelf a flat and narrow cheſt, which allows little play to the heart and lungs, he is naturally hypochondriac; but the reflection that this malady, being purely mechanical, cannot be cured, makes him free from anxiety in that reſpect: although he cannot cannot obviate the pain, yet he has refolution to be mafter of his thoughts and actions, fo as to difengage his attention from it, as if it did not concern him. This firmnefs he alfo recommends in cafes where perfons, efpecially thofe in bad health, are prevented from enjoying fleep, and who ought, as much as poffible, to divert their thoughts from the fenfation of their pain, or other caufe of anxiety, whether phyfical or mental, and direct them to fome object lefs interefting; and at the fame time to change their pofture in bed.

Kant ftrongly diffuades literary men from ftudy, while they are eating or walking—" if a perfon at the fame time fills the head and the belly, or confiderably exercifes the head and the feet; it will, in the former cafe, predifpofe him to hypochondriafis, and, in the latter, to epilepfy; it is neceffary to employ the mind and body alternately." Walking in the open air promotes health, as it diminifhes the attention, by dividing it among a variety of objects. On the contrary, when we walk, and at the fame time meditate deeply, or ftill more when we engage in ferious converfation with a companion, it is extremely fatiguing, and confequently prejudicial to health.

Kant having been troubled with a cold and cough, which frequently attacked him on going to bed, accuftomed himfelf to hold his lips clofe, and breathe only through the noftrils, which had the effect of preventing the attacks of this diforder; he therefore recommends this cuftom to perfons in general when they fleep, and alfo when they ride on horfeback; adding, that this practice promotes the fecretion of the faliva, which at once affifts digeftion, and encourages the neceffary evacuations.

Kant next intreats Hufeland to take particular care of his eyes, and complains of the modern ftyle of printing, as being extremely fatiguing to the fight. He cenfures the practice of reading rapidly, particularly running through a variety of newfpapers, journals, &c. Both Kant and Hufeland complain of the colour of the ink ufed in printing; which, inftead of being perfectly black, is generally almoft grey. Hufeland gives the Roman characters the preference, and, by way of joining example to precept, his '*Art of prolonging Life*,' in German, is printed in Roman letters—Kant prefers the German character.

There is not (fays Hufeland) a furer way to fhorten the thread of life, than to educate children in an anxious and delicate manner. Their organs and powers are by this means brought forward with fimilar precocity, as plants in a hot-houfe. An abufe of the fexual impulfe accelerates, in an extraordinary degree, old age and death. Thus, too early marriages are unfavourable to longevity: in our climates, the man fhould be twenty-four, and the woman eighteen, before they enter into the connubial ftate. Too violent exercife has has often proved fatal to youth: "how often have I ſeen a lovely woman's health ruined by an immoderate paſſion for dancing! I have ſeen the roſe ſuddenly fade, droop, and die."

The ſeaſon in which children are born is by no means a matter of indifference. The ſpring is more favourable to longevity than winter: the infant then reſpires more freely, is better able to enjoy the freſh air, and is leſs liable to be kept too cloſe and warm. The ſpring and ſummer have alſo a vivifying influence; and we ſee, that the animals born at that time are the moſt healthy and ſtout.

*Rules reſpecting the faculty of thinking.*

1. We ought not to devote ourſelves too much to ſerious thought, ſo as to neglect health: it is neceſſary ſometimes to exerciſe the mind, and ſometimes the body.

2. It is hurtful to reflect conſtantly upon the ſame ſubject; neither the mind nor the body ſhould be always engaged in the ſame exerciſe.

3. Metaphyſics and abſtract ideas are infinitely more fatiguing to the mind than other thoughts.

4. Do not always intenſely exert the mind in inventing or compoſing:— to read or tranſcribe occaſionally will give a ſalutary relief.

5. To engage the mind in ſtudy, at too early a period of life, impairs the conſtitution. Every mental exertion, before ſeven years of age, is injurious to the body.

6. Reflections to which the mind is leaſt diſpoſed, will ever be the moſt fatiguing.

7. To excite the mind by ſtimulants, ſuch as wine, opium, coffee, &c. is pernicious.—In caſe of neceſſity, however, a ſingle cup of coffee is the moſt adviſable expedient.

8. It is doubly prejudicial to health, to exerciſe the mind, while the ſtomach is employed in digeſtion. Thinking requires then extraordinary efforts, and renders the proceſs of digeſtion much more difficult.

9. We ought, as much as poſſible, to avoid reflection at the time of going to bed, or when we awake in the night.

10. It is improper, always to ſtudy in our chamber, and in a confined or inconvenient attitude: we ſhould occaſionally lye down, ſtand, or repair to the open air."

Among the things which ſhorten life, none has greater influence than fermented liquors of every kind—"it is ſwallowing liquid fire, which in a ſhocking degree accelerates the conſumption of vitality, and literally makes life a proceſs of combuſtion." The uſe of theſe liquors is not only productive of a variety of diſeaſes, but dreadfully impairs ſenſibility, both moral and phyſical; phyfical; and it may be remarked, that a perfon addicted to drinking fpirits is fo completely burnt up, as to be infufceptible of irritation; and, when he is attacked by difeafe, medicines have rarely the effect of affording him relief.

The author's remarks on the venereal diforder are truly terrific. " The fyphillitic virus combines every thing that is painful, loathfome, permanent and dreadful. Yet we laugh at this murderous poifon, and talk of it with pleafantry, as a fafhionable complaint. We fpeak as lightly of it as of a cough or coryza, and we generally neglect to oppofe its progrefs in our families with effectual remedies. No care is taken to check the rapid ftrides of this horrible contagion, which every where envenoms the vitals of fociety. My blood boils with indignation, when I fee it making its way into the country, and attacking the peafants, formerly fo healthy and robuft, who form that nucleus which fhould preferve manhood in vigour and primitive purity. This dreadful fcourge was, within thefe twenty years, not known even by by name, in villages where we now find two thirds of the inhabitants infected. I clearly forefee, that this evil will continue to extend itfelf, fo that no family will be free from its baneful influence : even the moft virtuous and auftere will be infected by nurfes, fervants, &c. It is time to form a barrier to oppofe this deftructive torrent; and I fee no other means for that end, than to eftablifh, upon more folid grounds, the empire of morality, efpecially among the higher claffes; to inftitute a good police of health, and diligently to inform the world of the nature of this poifon, its dangerous confequences, its fymptoms, and the beft means of prevention."

" The meafles and fmall-pox are certainly not *neceffary* evils: they may be avoided; and if general meafures were taken for that end, thofe loathfome diforders might undoubtedly be extirpated. The execution of this plan has been indeed already attempted, in fome countries; and the endeavours of the refpectable Profeffor Junker, to effect fo defirable a purpofe, are highly praife-worthy; but, as it cannot be expected, that this meafure will be univerfally adopted, while there are found even phyficians to oppofe it, it only remains to render the poifon as mild as poffible ; which experience fhews is beft effected by the practice of inoculation."

Mr. Hufeland proceeds to confider the poffibility of extirpating the fmall-pox, which he calculates might be effected in the courfe of four years, if, throughout the whole of civilized Europe, the infected were immediately prevented from communication with the healthy. Too much praife cannot be beftowed on thofe governments which have interefted themfelves to carry this this ſcheme into effect, particularly the ſouthern parts of Pruſſia. We cannot, however, but regret that there are governments which have refuſed to ſecond the philanthropic intentions of a man ſo zealous in the cauſe of humanity; and it is ſtill more diſtreſſing, that there are phyſicians ſo ſelfiſh as to deny their aſſiſtance to this benevolent plan, becauſe it would diminiſh the profits of their profeſſion. And, were an attempt to be made to exterminate the venereal diſeaſe, it would undoubtedly meet a ſtill great oppoſition, from ſimilar motives of intereſt.

Our limits will not allow us to extend farther the account of this intereſting work, and particularly to inſert the author's valuable practical maxims for the conduct of the aged. In a future number, we ſhall gratify our readers with this part of the treatiſe.

